# Effect of the Addition of Hulless Barley Flour on the Quality of Short-Dough Cookies

**DOI:** 10.3390/foods11162428

**Published:** 2022-08-12

**Authors:** Gjore Nakov, Marko Jukić, Gordana Šimić, Franjo Šumanovac, Daliborka Koceva Komlenić, Jasmina Lukinac

**Affiliations:** 1 Institute of Cryobiology and Food Technologies, Agricultural Academy—Sofia, 1407 Sofia, Bulgaria; 2 Faculty of Food Technology Osijek, Josip Juraj Strossmayer University of Osijek, 31000 Osijek, Croatia; 3 Agricultural Institute Osijek, 31000 Osijek, Croatia

**Keywords:** short-dough cookies, hulless barley, β-glucans, antioxidant capacity

## Abstract

Short-dough cookies are one of the most popular cereal-based products in the world, but usually they are considered as foods with a low nutritional value. Therefore, the aim of this study was to investigate the possibility of replacing part of the wheat flour (WF) with hulless barley flour (HLBF), in order to improve the functional properties and nutritional value of the cookies. Cookies were prepared from composite flours in the ratios HLBF:WF 0:100, 25:75, 50:50, 75:25, and 100:0. The results show that as the HLBF content in the composite flour increases, the viscosity of the corresponding HLBF:WF slurries also increases, due to the high dietary fiber content (especially the high β-glucan content), which is significantly higher when HLBF is included in the formulation. The addition of HLBF decreases the spread factor of the cookies, and increases their softness compared to the control cookies (100% WF), but these changes are not statistically significant up to 50% HLBF addition. The color of the HLBF:WF cookies is not significantly affected. A significant increase in total phenolic content (TPC) and increased antioxidant capacity (AOC) are observed in the HLBF:WF cookies. In addition, sensory evaluation confirms that WF can be replaced by up to 50% with HLBF without significant deterioration of the organoleptic properties of the cookies. It can be concluded that hulless barley serves as a promising raw material if the nutritional and functional properties of cereal-based products are to be improved.

## 1. Introduction

The quality characteristics of cookies depend on the chemical composition of the flour used and its quality. The process of cookie production depends on many factors: the raw materials used, the baking time, the type of cookies to be produced, etc. The most commonly used flour for cookie production is wheat flour (WF) [1]. Nowadays, there is an increasing interest in the use of non-traditional cereals (barley, oats, chia seeds, etc.) in cookie production. In addition, the newly produced cookies are considered to have better nutritional value, as various cereals are considered important sources of antioxidants (chemical compounds with high antioxidant activity) in the human diet [2]. Barley (*Hordeum vulgare* L.) is one of the oldest cultivated cereals, used for the production of beer, whiskey, yeast, substitute coffee, barley gruel, animal feed, and so on [3]. In recent years, there was increasing interest in the use of barley and barley flour in food production, due to the high content of dietary fiber such as β-glucan, B-complex vitamins, vitamin E (tocotrienols, and tocopherols), minerals, and phenolic compounds [4,5], which are believed to help prevent or alleviate various diseases such as heart disease, hypertension, diabetes, and obesity [6]. Many authors point out that barley grain and whole barley flour contain a high amount of β-glucans (2–11%), depending on genetic and environmental factors [7]. β-glucans are hydroscopic compounds that are soluble dietary fibers. They help in lowering blood cholesterol levels, and also have the ability to increase water-holding capacity [2]. Chemically, β-glucans are unsubstituted, unbranched polysaccharides, composed of β-D-glucopyranosyl monomers polymerized via (1,3)- and (1,4)-bonds, and can be divided into two types of polysaccharides, namely cellotriosyl and cellotetraosyl [8]. Recently, both the U.S. Food and Drug Administration (FDA) and the European Food Safety Authority (EFSA) approved the health claims for barley and barley β-glucan, so that they can be considered as “functional foods” [9,10]. The intake of bioactive compounds and dietary fiber through the consumption of barley-containing products (cookies, bread, cakes, etc.) isa way to meet the human body’s need for these compounds [4,11].

The majority of barley production consists of the cultivation of covered (hulled) varieties used for malt and feed. The problem with using this type of barley in food is its unpalatable hull. Therefore, barley is often pearled, which means that in addition to the outer hull, part of the bran is also removed, reducing the micronutrient and mineral content of the barley flour and, thus, reducing its nutritional value [12]. In view of the above, it can be assumed that the use of hulless barley in food can also bring certain economic savings, as it is easier to use compared to hulled barley varieties, which require additional operations before they are suitable for use in food production. Therefore, recently more and more attention was paid to the development of new varieties of hulless (naked) barley that can be more easily incorporated into food products. Studies investigating the influence of barley flour on cereal products (bread, pasta, cookies) have mostly been conducted with hulled barley, and very few investigated the influence of hulless barley, especially the influence on cookie quality. Until now, only a few such studies have been conducted [13,14,15,16]. Narwal et al. evaluated the effect of HLBF supplementation on AOC, total phenolic, and β-glucan content, and the dimensional and sensory properties of cookies, and found that 30% HLBF supplementation is an excellent way to improve the nutritional and functional properties of cookies [13]. Aly et al. found that 40% HLBF supplementation significantly increases AOC, and causes color changes in the cookies, but sensory scores decrease slightly when 40% HLBF is incorporated into the cookie formulation [16]. In the study by El-Hadidy et al., only the 25% HLBF addition was investigated, and sensory values are even higher in these samples than in the 100% wheat cookies [14]. The most comprehensive study to date on the influence of replacing WF with HLBF in the production of cookies was conducted by Abdelazim et al. In their study, they investigated the influence of replacing WF (25, 50, 75, and 100%) with the flour of three varieties of hulless barley, and found that although the sensory characteristics of the cookies deteriorate compared to the control WF cookies, HLBF can be used to improve the nutritional value of the cookies. However, they did not use the whole grain HLBF, but HLBF with an extraction level of 72% [15]. All these studies used different barley varieties, different levels of HLBF substitution, and different cookie formulations. Therefore, any new study dealing with the influence of using HLBF in cookie production can be considered as a significant contribution to completing the knowledge about the possibility of using hulless barley in cookie production.

The aim of this study was to investigate the effects of using flour from the hulless barley variety “Osvit” in the production of cookies from composite flours containing hulless barley flour (HLBF) and WF at substitution levels of 25, 50, 75, and 100%. The pasting properties of the composite flours, chemical composition (including fiber and β-glucan content) and energy value, dimensions, texture, sensory properties, and AOC of HLBF:WF composite cookies were evaluated to determine the maximum proportion of HLBF that can replace WF so that the physical and sensory properties of the cookies do not deteriorate, while improving their nutritional value and increasing their functional properties.

## 2. Results and Discussion

### 2.1. Pasting Properties

In this study, 100% commercial plain WF (control), 100% whole grain HLBF, and three different HLBF:WF composite flours (25:75, 50:50 and 75:25) were used to produce HLBF:WF composite cookies. To investigate pasting properties of the HLBF:WF slurries, the Brabender Micro Visco-Amylo-Graph was used.

Table 1 shows the results of the five viscosity parameters considered. The pasting temperature increases, although it is not statistically significant (*p* < 0.05), with the increase in the percentage of barley flour in the mixture (from 60.8 °C for 100% WF to 61.6 °C for 100% HLBF). The lowest peak viscosity is observed at 100% WF (897.0 BU). With the increase in HLBF content in the blend, the peak viscosity increases to 1254.3 BU in 100% HLBF. A higher peak viscosity indicates a greater ability of the starch granules to bind water, and reflects the swelling ability of the granules before breakdown [17]. The breakdown viscosity also increases with increasing HLBF content (from 318.7 BU for 100% WF to 546.7 BU for 100% HLBF), as well as setback viscosity (from 437.3 BU for 100% WF to 582.7 BU for 100% HLBF), indicating a better retrogradation ability of the samples with higher HLBF content. There are no statistically significant differences between the samples with respect to peak temperature (*p* < 0.05). A similar pattern of pasting properties of composite flours with HLBF and WF is observed in the study by Liu et al. [18]. These results are also in agreement with the results of Sharma and Gujral. They concluded that the main reason for the increase in viscosity when the amount of barley (hulled) flour in the blend is increased could be the high soluble fiber content in the barley flour [4]. Since β-glucan is the main component of soluble fiber in barley, Gamel et al. considered that β-glucan has a great influence on increasing the paste viscosity. β-glucans generally bind free water present in the system and, at the same time, increase the viscosity of suspensions containing them [11,19].

### 2.2. Physical Characteristics of HLBF:WF Cookies

The physical characteristics of cookies are one of the key factors when customers decide whether to buy them or not. Table 2 summarizes the results of the effects of HLBF on the dimensions of the cookies and their textural properties. From the results presented, it can be seen that increasing the HLBF content in a composite flour results in a significant decrease in cookie width and cookie thickness. Cookie width significantly decreases from 6.76 cm for 100% WF to 6.10 cm for 100% HLBF cookies (*p* < 0.05), and thickness decreases from 1.28 cm for 100% WF to 1.22 cm for 100% HLBF cookies, but this reduction is not statistically significant (*p* < 0.05). As the cookie width decreases much more than the cookie thickness, the spread factor of the cookies also decreases by 5% (from 52.80 for 100% WF cookies to 50.14 for 100% HLBF cookies). A similar decrease in cookie spread factor is observed in the study by Elhadidy et al., when 25% of WF is replaced with HLBF [14]. Sharma and Gujral pointed out in their study that the complete replacement of wheat flour with barley flour results in a 31% reduction in the spread factor of the cookies [4]. This reduction is significantly higher than in our study, but in contrast to our results, the thickness of the cookies is increased in Sharma and Gujral’s study, and since thickness is reciprocal to spread factor, it significantly affects the reduction in spread factor. A reduction in the spread of cookies by increasing the amount of barley in the composite flour for cookies production is also found in some other studies [13,20,21,22]. In addition, Lee et al. report that one of the reasons for the reduction in the spread factor of cookies is β-glucans, which are normally found in oats and barley [23].

The reduction in the width of the cookies and, consequently, of the spread factor, can be explained by the higher dough viscosity in samples with increased HLBF content. Mancebo et al. reached a similar conclusion in their study on the effects of different non-wheat flours on the quality of gluten-free cookies [24]. This is likely due to the fact that higher dietary fiber (β-glucan) content in HLBF increases the water-holding capacity of these flours, and reduces the amount of water available for sugar dissolution, which is an important factor in cookie spreading during baking. β-glucan exhibits the properties of hydrocolloids and, thus, mimics the gluten behavior known to reduce cookie spreading.

The determination of the textural properties of HLBF:WF composite cookies was performed using the three-point bend/break test. The results are also shown in Table 2. As for the decreased spread factor, the reduced dissolution of sugar, caused by the increased water binding of HLBF β-glucans, may also explain the deteriorated textural properties of the HLBF:WF cookies compared to the control WF cookies. As can be seen in Table 2, with the increasing addition of HLBF, the snapping force decreases (from 106.11 N for 100% WF to 95.25 N for 100% HLBF cookies) and the breaking distance decreases (from 1.82 mm for 100% WF to 3.61 mm for 100% HLBF cookies). Consequently, the bending force index is significantly (*p* < 0.05) reduced (from 58.30 N/mm for 100% WF to 26.39 N/mm for 100% HLBF cookies), due to a much softer, crumbly texture of the HLBF cookies with increased graininess. These results, showing the softening effect of barley flour in HLBF:WF cookies and the reduced cohesion of these cookies, are in agreement with several other studies that investigated the replacement of WF with barley or barley malt flour [15,20,21,25,26], as well as the addition of barley and oat β-glucan-rich flour fractions in a cookie recipe [22,23]. All these results indicate that dietary fiber in barley flour plays an important role in the dimensional and textural properties of cookies, by interfering with the sucrose–water matrix.

### 2.3. Color of HLBF:WF Cookies

In addition to the amount of sugar prescribed by the recipe, as well as the temperature and baking time, the color of the cookies is mainly influenced by the type of flour used and the amount of pigments it contains.

**Figure 1:**
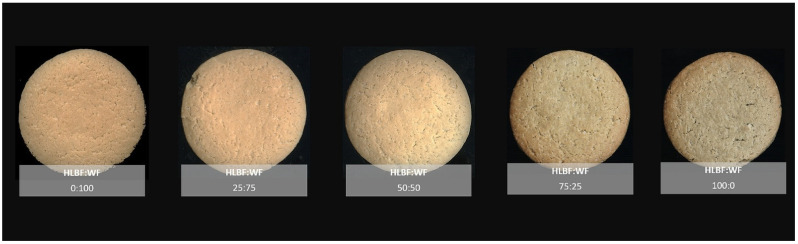
The composite cookies containing wheat flour (WF) and hulless barley flour (HLBF).

The color of the different cookies, determined by the CIE*Lab* coordinates, is shown in Table 3. From the results presented, it can be concluded that the baking process results in a significant decrease in L* (lightness) (from 66.18 for 100% WF to 62.90 for 100% HLBF cookies), a significant increase in a* (redness) (from 5.49 for 100% WF to 7.48 for 100% HLBF cookies), and a decrease in the color parameter b* (yellowness) (from 36.51 for 100% WF to 31.68 for 100% HLBF cookies) (*p* < 0.05). The same direction of color changes are observed in the studies by Žilić et al. [27] and by Frost et al. [21], when wheat flour is replaced by whole grain barley flour. Although the color changes during baking are due to the influence of the Maillard reaction and the caramelization of the sugar, the differences in the color of the cookies with increasing amounts of HLBF in the recipe result from the different colors of WF and HLBF. The color of HLBF is significantly influenced by anthocyanins present in the pericarp and aleurone layer of hulless barley, which cause the purple–blue hues of HLBF [28]. This is consistent with our results showing an increase in a* and a decrease in b* values when WF is replaced by HLBF. Increased a* values indicate a stronger red hue and decreased b* values indicate a stronger blue hue, which, in combination, results in a slightly purple coloration of the cookies. Although the differences in CIE*Lab* parameters between HLBF:WF composite cookies are mostly statistically significant (*p* < 0.05), these differences are actually very difficult to detect. This is evident from the calculated color differences (*ΔE*) with respect to the control cookies with 100% WF. Only the sample with 100% HLBF has *ΔE* > 5, which is the smallest color difference that an average consumer can perceive [29]. Images of composite HLBF:WF cookies are shown in Figure 1.

### 2.4. Proximate Composition, Water Activity (a_w_), and Energy Value of HLBF:WF Cookies

The proximate composition of HLBF:WF cookies is presented in Table 4. By increasing the amount of HLBF incorporation, the protein content significantly (*p* < 0.05) increases from 6.03 to 7.02%, available carbohydrates decreases from 71.25 to 65.03%, and ash content increases from 0.78 to 1.47% for 100% WF and 100% HLBF cookies, respectively. The fat content does not vary much because the majority of fat originates from added shortening. The decrease in the content of available carbohydrates can be attributed to the fact that the addition of HLBF increases the content of crude fiber. These results are consistent with other studies on the effects of replacing WF with HLBF in cookie formulation [15,16]. The calculated energy value for the control cookies (100% WF) is 446.78 kcal/100 g, which shows that the cookies have a high energy-to-weight ratio. The energy value is significantly (*p* < 0.05) decreased with increasing HLBF supplementation, and the 100% HLBF cookies have a 4.16% lower energy value than the control 100% wheat cookies. El-Hadidy et al. report that there is no change in the energy value when 25% of WF is replaced with flour of HLBF [14].

Both moisture content and water activity increases when HLBF is incorporated into the cookie formulation (Table 4). Moisture content increases from 4.61% for 100% WF to 5.99 for 100% HLBF cookies, and water activity increases from 0.41 for 100% WF to 0.53 for 100% HLBF cookies. Despite the significant increase in water activity, it is still below the value of 0.61, which is considered the lower limit for any microbial growth [30]. The same increase is obtained in some other studies when barley flour is used as a partial or total substitute for WF in the production of cookies [4,21,22]. The main reason for this increase is the higher dietary fiber (DF) content of HLBF cookies, as fiber has the ability to retain water at high temperatures, such as during cookie baking [21].

The highest content of total (TDF), insoluble (IDF), and soluble (SDF) was determined in cookies with complete replacement of WF by HLBF (9.84%, 5.24%, and 4.60%, respectively). According to the Annex of Regulation (EC) No. 1924/2006, barley grain fiber contributes to an increase in fecal bulk, and can be considered a “high fiber source” food if it contains more than 6 g/100 g of dietary fiber [31]. The incorporation of 50% HLBF in the cookie formulation meets this condition, and these cookies can be considered a functional food. In addition, the EFSA Panel issued a scientific opinion on reference values for carbohydrates and dietary fiber, stating that an intake of 25 g of dietary fiber per day is sufficient for normal laxative performance in adults [32]. One cookie with 50% HLBF replacement (about 24 g) in our study contains 1.50% dietary fiber, so a person can achieve 24% of the recommended daily value for dietary fiber by eating just four cookies. It is important to note that hulless barley generally has less IDF and, consequently, less TDF than covered barley varieties because the hull of covered barley is mainly composed of IDF [33]. In our study, the ratio of IDF to SDF in HLBF:WF cookies is about 60:40, which is considered a well-balanced ratio of dietary fiber in terms of health benefits [34,35]. SDFs are thought to be responsible for reducing the activity of intestinal enzymes, and lowering postprandial glucose levels and glycemic response. Since SDFs are highly susceptible to fermentation, they also induce increased production of short-chain fatty acids (SCFAs), which are known to reduce the risk of cardiovascular diseases [36]. The main component of SDF in barley, as in hulless barley, is β-glucans. As the β-glucan content in WF is very low, the control 100% WF sample contains only 0.12% β-glucans. The β-glucan content in HLBF:WF cookies is significantly higher (0.77, 1.42, 2.14, and 2.80% in cookies with 25, 50, 75, and 100% HLBF added, respectively) (*p* < 0.05). An increase in β-glucan content in cookies is also observed in other studies investigating the replacement of WF with barley flour [13,20,37]. Recently, the importance of using barley as a “functional food” in human nutrition was recognized, especially after the approval of health claims for barley and barley β-glucan by the European Food Safety Authority (EFSA) and the US Food and Drug Administration (FDA) [9,10]. However, according to Commission Regulation (EU) No. 432/2012, the quantified portion of a food should contain 1 g of β-glucans to meet the claim for maintaining normal blood cholesterol levels [38]. The amounts of β-glucans in our study ranges from 0.23–0.84 g per qualified portion, which is equivalent to 30 g (approximately one and a half cookies). Therefore, these cookies cannot be labeled with such health claims, but it is important to emphasize that a person eating about seven cookies per day can achieve the beneficial effect of consuming β-glucans, which is 3 g per day according to the same regulation.

### 2.5. Total Phenolic Content (TPC) and Antioxidant Capacity (AOC) of HLBF:WF Cookies

In addition to the significant dietary fiber content, the nutritional value of hulless barley is also complemented by the high content of phenolic substances and the associated high AOC [39]. Consequently, cookies with added HLBF have significantly higher TPC and higher AOC compared with 100% WF cookies (Table 5). The TPC increases from 160.53 µg GAE/g for 100% WF to 621.42 µg GAE/g for 100% HLBF cookies. Several other researchers also report that increasing the barley flour content in the cookie formulation also increases the TPC [4,13,20]. The AOC (DPPH scavenging activity) of HLBF:WF composite cookies increases from 2.09 mmol TE/100 g for 100% WF to 2.34 mmol TE/100 g for 100% HLBF cookies. However, the AOC of cookies does not only depend on the phenolic compounds, but can also be modified by the baking process through the development of caramelization and Maillard reaction products. Therefore, it is of great importance to determine the AOC of the final products and not only of the raw material.

Narwal et al. also report that as the amount of HLBF in the cookie composition increases, the AOC also increases [13]. The same results are obtained in the study by Sharma et al., when flour from covered barley is used for cookie production [4]. In contrast to these results, Gupta et al. report an increase in AOC in cookie dough when the proportion of barley flour in the recipe is increased, but there is no increase in AOC in baked cookies. This effect is explained by possible degradation of barley proanthocyanidins during baking [20].

### 2.6. Sensory Evaluation

The sensory ratings of the HLBF:WF composite cookies, as determined by sensory analysis using a nine point hedonic scale, are summarized in Table 6. The results show that the color scores of the HLBF:WF cookies are lower with increasing HLBF addition, but the color changes are not statistically significant (*p* < 0.05). This also confirms the results of the instrumental color determination, when only the 100 HLBF cookies have *ΔE* > 5 (6.16) compared to a control 100 WF cookie, which is very difficult for the average consumer to detect. Only a few evaluators mentioned that this sample has a slightly darker color, which negatively affects the rating. Panelists perceive the cookies with the higher spread factor as more acceptable, and give a significantly lower rating (5.9) for shape to the 100% HLBF cookie (*p* < 0.05). The most significant differences between the HLBF:WF composite cookies are observed for texture. The sample with 50% HLBF added has a score of 7.1, which is lower than the score for the control 100% WF cookies, but not significantly different (*p* < 0.05). The panelists note that this sample is softer than the control sample, but this does not significantly affect the score. Samples with 75% and 100% HLBF receive significantly lower ratings due to the highlighted crumbliness. There are no significant differences among samples for odor scores. The sample with 25% HLBF has the highest score for taste (7.7). Only the cookie with 100% HLBF receives a statistically lower score (6.2) compared to the control cookie with 100% WF (7.6) (*p* < 0.05). Overall acceptability ratings are 7.8, 7.7, and 7.3 for 100% WF, 25:75 HLBF:WF, and 50:50 HLBF:WF composite cookies, respectively. These ratings place these samples between “like very much” and “like moderately” on the nine point hedonic scale. The overall score for cookies with 75% and 100% HLBF addition is 6.7 and 6.2, respectively, which can be described as a score between “like moderately” and “like slightly”.

Sharma et al. reported a similar decrease in sensory ratings of cookies with increasing barley flour addition, but compared to our study, higher scores were obtained for overall acceptability of cookies with the addition of 100% barley flour [4]. This sample scored 7.1 points on the 9-point hedonic scale, which is higher than the score for 100% HLBF cookies in our study, which scored 6.2 points. According to consumer sensory analysis conducted by 100 consumers in a study by Frost et al., a cookie sample with 30% barley flour added had a score of 6.8, which was the highest score among all samples and slightly higher than the score for the control cookie with 100% WF (6.7) and the score for the sample with 50% barley flour (6.3). They concluded that replacing wheat flour with barley flour up to 50% is sensory acceptable. Further increase in barley flour addition decreased the sensory scores of the cookies down to 5.8 points for cookies with 70% barley flour [21].

## 3. Materials and Methods

### 3.1. Materials

In this study, commercial all-purpose WF (Tena-Žito Ltd., Đakovo, Croatia) from a previous study was used [25]. WF contained 11.81% protein, 79.60% starch, 1.54% fat, 0.57% ash, 6.48% TDF, 3.94% IDF, 2.54% SDF, and 0.22% β-glucan (dry matter basis). The hulless barley variety “Osvit” (Agricultural Institute Osijek, Croatia) was also previously used [39]. The whole grain HLBF was prepared by milling barley grains in laboratory mill IKA MF-10 (IKA-Werke GmbH & Co., Staufen, Germany) using the 1 mm sieve. The obtained HLBF contained 13.93% protein, 63.47% starch, 2.42% fat, 1.96% ash, 18.22% TDF, 9.71% IDF, 8.51% SDF, and 5.18% β-glucan (dry matter basis). The other ingredients for cookie production were from a local market: shortening (Zvijezda Ltd., Zagreb, Croatia), sucrose, sodium bicarbonate (NaHCO_3_), and sodium chloride (NaCl).

### 3.2. Methods

#### 3.2.1. Pasting Properties of HLBF:WF Composite Flours

The Micro Visco-Amylo-Graph (Brabender OGH, Duisburg, Germany) was used to evaluate the pasting properties of composite HLBF:WF flours. A suspension containing 15 g of flour (14% w.b.) and 100 mL of distilled water was added to a mixing bowl of the instrument and heated from 30 to 92 °C at a rate of 5 °C/min, held constant at 92 °C for 5 min, cooled to 50 °C at a rate of 5 °C/min, and finally held at 50 °C for 1 min. The rate of 250 min^−1^ was constant throughout the analysis. Pasting and peak temperatures, peak, breakdown, and setback viscosities were recorded. Viscosity parameters were expressed in Brabender units (BU). Three replicate measurements were made for each sample.

#### 3.2.2. Production of HLBF:WF Composite Cookies

The cookies were prepared according to the AACC international method 10–50.05 [40]. The exact formulations of composite HLBF:WF cookies can be found in Table 7. An electronic mixer (Gorenje MMC800W, Slovenia) was used to mix the ingredients into a dough, and round pieces of dough (60 mm in diameter and 7 mm thick) were placed in a convection oven (Wiesheu Minimat Zibo, Wiesheu GmbH, Germany) and baked at 205 °C for 12 min. The cookies were made in triplicate batches.

#### 3.2.3. Physical Characteristics of HLBF:WF Cookies

The dimensions of the cookies (width (W) and thickness (T)) were determined using the instructions of given in the AACC international method 10 50.05 [40]. The spread factor was calculated as W/T multiplied by 10. Six cookies from each batch were measured.

The TA.XT2i Texture Analyzer (Stable Microsystems Ltd., Surrey, UK) was used to perform the three-point bend/break test. The distance between two lower supports was 40 mm, and the test speed of the knife blade was 1 mm/s. The snapping force (N) and the breaking distance (mm) were obtained from the test curve. By dividing these two parameters, the bending force index (N/mm) was calculated. This index served as an indicator of the hardness/softness of the cookies [25]. Two cookie samples were tested from each batch.

The CR-400 chromameter (Konica Minolta, Japan) was used to determine the color of the cookie surface. The CIEL*a*b* color model was used. The L* value (from 0 to 100) represents brightness (lightness) or luminance, the b* value (−128 to 127) represents blue–yellow, and the a* value (−128 to 127) represents the green–red axes of the color space. The CIE76 color difference equation was used to calculate the total color difference (*ΔE*) between the composite HLBF:WF cookies and the control cookie (100% WF).

#### 3.2.4. Chemical Properties of HLBF:WF Cookies

Moisture content was determined in accordance with AACC international method 44-15.02 [40] and water activity was measured using the Hygropalm AW1 portable indicator (Rotronic, Bassersdorf, Switzerland) in AwQuick mode. The proximate composition of the cookie samples (protein, fat, and ash content) was determined according to official methods of analysis of the Association of Official Analytical Chemists (AOAC) [41]. Carbohydrates were expressed as available carbohydrates, and their amount was calculated by subtracting the moisture, protein, fat, ash, and insoluble fiber content from 100. The Atwater formula was used to calculate the energy value: Energy (kcal/100 g) = protein (g/100 g) × 4 + fat (g/100 g) × 9 + carbohydrate (g/100 g) × 4. Energy (kJ/100 g) = energy (kcal/100 g) × 4.184.

AACC international method 32-07.01 (Megazyme assay procedure K-TDFR) was used for determination of the dietary fiber content (TDF, NDF, and IDF), and AACC international method 32-23.01 (Megazyme assay procedure for cooked, toasted, or extruded cereal products—streamlined method K-BGLU (McCleary method)) was used for the determination of β-glucan [40].

For the determination of TPC and AOC, cookies were ground (Grindomix GM 200, Retsch GmbH, Haan, Germany) and 1 g of the sample was extracted in 10 mL of 80% methanol, vortexed for 1 min, and shaken for 2 h at 150 rpm on an electronic shaker. The mixture was centrifuged at 3000× *g* for 10 min, and the supernatant was used for further TPC and AOC analyzes. TPC was determined according to the method of Singleton and Rossi [42], and AOC (DPPH scavenging activity) according to the method of Brand-Williams et al. [43]. Calibration curves were prepared with Trolox reagent (6-hydroxy-2,5,7,8-tetramethylchroman-2-carboxylic acid), and results were expressed as Trolox equivalent (mmol TE/100 g).

#### 3.2.5. Sensory Evaluation

A panel of ten evaluators conducted the sensory analysis of the cookie samples. All were staff and students of the Department of Cereal Technology at the Faculty of Food Technology Osijek, Croatia, and all had previous experience with sensory analysis. There were seven women and three men in the panel (median age = 24 years). The inclusion criteria for the evaluators were the absence of health problems that could affect the sensory evaluation (anosmia, color blindness, etc.), and the usual preference for consuming similar types of cookies. Sensory analysis was performed in a tasting room equipped with individual test booths. The panelists were given a brief introduction to give them an insight into the research and the samples to be tested. All five cookie samples were handed to the evaluators at the same time and they were asked to rinse their mouths with water between tastings. The 9 point hedonic scale was used to assess sensory properties. Scores from 1 to 9 were: extremely dislike (1), very much dislike (2), moderately dislike (3), slightly dislike (4), neither dislike nor like (5), slightly like (6), moderately like (7), very much like (8), and extremely like (9).

#### 3.2.6. Statistical Analysis

Analysis of variance (ANOVA) followed by Tukey’s honestly significant difference (HSD) test was used to detect differences between samples (*p* < 0.05). The statistical add-in for Microsoft Excel XLSTAT software (Addinsoft, New York, NY, USA) was used for statistical analysis of experimental data.

## 4. Conclusions

Barley has many beneficial health properties, due to increased dietary fiber content (especially high β-glucan), and high TPC and AOC. Therefore, it can be expected that the use of barley flour in the production of cookies increases the nutritional value and improves the functional properties. Since covered (hulled) barley has an unpalatable hull that can deteriorate the properties of cookies when whole grain flour is used or when the barley is pearled before milling into flour, which reduces the nutritional value of barley flour, hulless barley is imposed as an excellent raw material for flour production that can be used in cookie production, and give them both nutritional value and sensory acceptable properties. With the increasing replacement of wheat flour by barley flour, the physicochemical properties of the cookies change significantly. The spread factor and the hardness of the cookies decrease, and the color becomes slightly darker. From a nutritional and health point of view, it is particularly important that the amount of dietary fiber, especially β glucan, increases, as well as the TPC and AOC. The results show that by replacing WF with up to 50% HLBF, there are no significant differences between HLBF:WF composite cookies and 100% WF cookies in terms of physical and sensory properties, but the nutritional and functional properties are improved.

## Figures and Tables

**Table 1:** Pasting properties of composite flours containing wheat flour (WF) and hulless barley flour (HLBF).

HLBF:WF	Pasting Temperature (°C)	Peak Viscosity (BU)	Peak Temperature (°C)	Breakdown Viscosity (BU)	Setback Viscosity (BU)
0:100	60.8 ^a^ ± 0.2 ^1^	897.0 ^e^ ± 2.8	89.4 ^a^ ± 0.2	318.7 ^d^ ± 1.0	437.3 ^d^ ± 7.0
25:75	61.0 ^a^ ± 0.1	1044.3 ^d^ ± 7.8	89.3 ^a^ ± 0.3	434.3 ^c^ ± 3.7	492.0 ^c^ ± 8.7
50:50	61.3 ^a^ ± 0.4	1088.7 ^c^ ± 23.3	89.3 ^a^ ± 0.1	440.0 ^c^ ± 8.4	517.3 ^bc^ ± 18.9
75:25	61.5 ^a^ ± 0.4	1136.0 ^b^ ± 14.6	89.2 ^a^ ± 0.4	469.3 ^b^ ± 7.9	554.3 ^ab^ ± 12.7
100:0	61.6 ^a^ ± 0.8	1254.3 ^a^ ± 6.3	89.1 ^a^ ± 0.1	546.7 ^a^ ± 4.0	582.7 ^a^ ± 9.9

^1^ The values are mean ± SD (*n* = 3). Mean values in the same column with different superscript letters (a–e) are significantly different (*p* < 0.05).

**Table 2:** Dimensional and textural properties of composite cookies containing wheat flour (WF) and hulless barley flour (HLBF).

HLBF:WF	Width (cm)	Thickness (cm)	Spread Factor	Snapping Force (N)	Breaking Distance (mm)	Bending Force Index (N/mm)
0:100	6.76 ^a^ ± 0.07 ^1^	1.28 ^a^ ± 0.07	52.80 ^b^ ± 0.02	106.11 ^a^ ± 2.23	1.82 ^b^ ± 0.33	58.30 ^a^ ± 2.32
25:75	6.67 ^ab^ ± 0.23	1.26 ^a^ ± 0.07	52.98 ^a^ ± 0.02	106.26 ^a^ ± 3.51	2.21 ^ab^ ± 0.41	48.08 ^b^ ± 3.49
50:50	6.47 ^abc^ ± 0.12	1.23 ^a^ ± 0.14	52.43 ^c^ ± 0.07	103.53 ^ab^ ± 3.27	3.44 ^a^ ± 0.61	30.10 ^c^ ± 3.10
75:25	6.19 ^bc^ ± 0.16	1.23 ^a^ ± 0.07	50.54 ^d^ ± 0.03	98.11 ^ab^ ± 4.62	3.46 ^a^ ± 0.48	28.36 ^c^ ± 2.15
100:0	6.10 ^c^ ± 0.17	1.22 ^a^ ± 0.03	50.14 ^e^ ± 0.08	95.25 ^b^ ± 2.16	3.61 ^a^ ± 0.52	26.39 ^c^ ± 1.93

^1^ The values are mean ± SD (*n* = 6). Mean values in the same column with different superscript letters (a–e) are significantly different (*p* < 0.05).

**Table 3:** Color of cookies made from composite flours containing wheat flour (WF) and hulless barley flour (HLBF).

HLBF:WF	L*	a*	b*	*ΔE*
0:100	66.18 ^a^ ± 0.33 ^1^	5.49 ^d^ ± 0.32	36.51 ^a^ ± 0.53	-
25:75	64.99 ^b^ ± 0.16	6.05 ^cd^ ± 0.41	35.14 ^b^ ± 0.26	1.90
50:50	64.07 ^c^ ± 0.29	6.59 ^bc^ ± 0.20	33.96 ^c^ ± 0.46	3.49
75:25	63.43 ^d^ ± 0.30	7.07 ^ab^ ± 0.37	32.77 ^d^ ± 0.22	4.90
100:0	62.90 ^d^ ± 0.17	7.48 ^a^ ± 0.33	31.68 ^e^ ± 0.37	6.16

^1^ The values are mean ± SD (*n* = 6). Mean values in the same column with different superscript letters (a–e) are significantly different (*p* < 0.05).

**Table 4:** Proximate composition, water activity (*a_w_*), total, insoluble, and soluble dietary fiber (TDF, IDF, SDF) content, and energy value of composite cookies containing wheat flour (WF) and hulless barley flour (HLBF).

Parameter	HLBF:WF				
	0:100	25:75	50:50	75:25	100:0
Protein (%. w.b.)	6.03 ^d^ ± 0.12 ^1^	6.33 ^cd^ ± 0.06	6.55 ^bc^ ± 0.09	6.77 ^ab^ ± 0.07	7.02 ^a^ ± 0.10
Carbohydrates (%. w.b.)	71.25 ^a^ ± 0.05	69.92 ^b^ ± 0.13	68.39 ^c^ ± 0.08	66.70 ^d^ ± 0.04	65.03 ^e^ ± 0.12
Fat (%. w.b.)	15.30 ^bc^ ± 0.03	15.53 ^a^ ± 0.06	15.58 ^a^ ± 0.04	15.58 ^a^ ± 0.04	15.56 ^a^ ± 0.09
Ash (%. w.b.)	0.78 ^e^ ± 0.02	0.95 ^d^ ± 0.01	1.12 ^c^ ± 0.03	1.32 ^b^ ± 0.01	1.47 ^a^ ± 0.03
Moisture (%)	4.61 ^d^ ± 0.07	4.63 ^cd^ ± 0.07	4.89 ^cd^ ± 0.06	5.35 ^b^ ± 0.05	5.99 ^a^ ± 0.14
Water activity	0.41 ^c^ ± 0.01	0.43 ^bc^ ± 0.03	0.46 ^abc^ ± 0.03	0.50 ^ab^ ± 0.02	0.53 ^a^ ± 0.02
TDF (%. d.b.)	3.50 ^e^ ± 0.07	4.37 ^d^ ± 0.04	6.25 ^c^ ± 0.16	8.02 ^b^ ± 0.09	9.84 ^a^ ± 0.19
IDF (%. d.b.)	2.13 ^e^ ± 0.11	2.75 ^d^ ± 0.11	3.63 ^c^ ± 0.22	4.50 ^b^ ± 0.10	5.24 ^a^ ± 0.16
SDF (%. d.b.)	1.37 ^d^ ± 0.09	1.63 ^d^ ± 0.14	2.62 ^c^ ± 0.16	3.52 ^b^ ± 0.07	4.60 ^a^ ± 0.25
β-glucan (%. d.b.)	0.12 ^e^ ± 0.03	0.77 ^d^ ± 0.05	1.42 ^c^ ± 0.08	2.14 ^b^ ± 0.12	2.80 ^a^ ± 0.10
Energy value (kJ/100 g) (kcal/100 g)	1869.33 (446.78)	1860.97 (444.78)	1840.75 (439.95)	1816.55 (434.17)	1791.49 (428.18)

^1^ The values are mean ± SD (*n* = 3). Mean values in the same row with different superscript letters (a–e) are significantly different (*p* < 0.05).

**Table 5:** Total phenolic content (TPC) and antioxidant capacity (AOC) of cookies made from composite flours containing wheat flour (WF) and hulless barley flour (HLBF).

HLBF:WF	Total Polyphenols (µg GAE/g DW)	DPPH Scavenging Activity (mmol TE/100 g)
0:100	160.53 ^e^ ± 2.71 ^1^	2.09 ^b^ ± 0.12
25:75	270.51 ^d^ ± 1.71	2.15 ^ab^ ± 0.02
50:50	401.07 ^c^ ± 3.41	2.23 ^ab^ ± 0.04
75:25	521.54 ^b^ ± 2.12	2.29 ^ab^ ± 0.05
100:0	621.42 ^a^ ± 4.12	2.34 ^a^ ± 0.06

^1^ The values are mean ± SD (*n* = 3). Mean values in the same column with different superscript letters (a–e) are significantly different (*p* < 0.05).

**Table 6:** Sensory scores of composite cookies containing wheat flour (WF) and hulless barley flour (HLBF).

HLBF:WF	Color	Shape	Texture	Odor	Taste	Overall
0:100	7.9 ^a^ ± 1.0 ^1^	7.7 ^a^ ± 0.6	8.0 ^a^ ± 1.1	7.6 ^a^ ± 1.4	7.6 ^a^ ± 0.5	7.8 ^a^ ± 0.4
25:75	7.9 ^a^ ± 1.5	7.7 ^a^ ± 0.9	7.8 ^a^ ± 1.2	7.6 ^a^ ± 0.9	7.7 ^a^ ± 1.1	7.7 ^a^ ± 0.4
50:50	7.4 ^a^ ± 1.2	7.4 ^a^ ± 1.1	7.1 ^ab^ ± 1.0	7.3 ^a^ ± 1.2	7.5 ^a^ ± 1.2	7.3 ^a^ ± 0.4
75:25	7.2 ^a^ ± 1.6	6.8 ^ab^ ± 1.2	5.6 ^bc^ ± 1.1	7.1 ^a^ ± 1.8	7.0 ^ab^ ± 1.5	6.7 ^ab^ ± 0.6
100:0	6.9 ^a^ ± 1.3	5.9 ^b^ ± 0.7	5.1 ^c^ ± 1.1	7.0 ^a^ ± 1.1	6.2 ^b^ ± 1.3	6.2 ^b^ ± 0.5

^1^ The values are mean ± SD (*n* = 10). Mean values in the same column with different superscript letters (a–c) are significantly different (*p* < 0.05).

**Table 7:** Formulation of composite cookies containing wheat flour (WF) and hulless barley flour (HLBF) (g).

HLBF	WF	Shortening	Sucrose	NaCl	NaHCO_3_	6% Dextrose Solution	Water
-	100	28.4	57.8	0.9	1.1	14.7	7.1
25	75	28.4	57.8	0.9	1.1	14.7	7.1
50	50	28.4	57.8	0.9	1.1	14.7	7.1
75	25	28.4	57.8	0.9	1.1	14.7	7.1
100	-	28.4	57.8	0.9	1.1	14.7	7.1

## Data Availability

Data is contained within the article.
